# Action fluency and conscious intention: being aware of what we are about to do

**DOI:** 10.1093/cercor/bhag108

**Published:** 2026-08-10

**Authors:** Zheng Huang, Silvia Seghezzi, Patrick Haggard

**Affiliations:** Institute of Cognitive Neuroscience, University College London, 17 Queen Square, London, WC1N 3AZ, United Kingdom; Institute of Cognitive Neuroscience, University College London, 17 Queen Square, London, WC1N 3AZ, United Kingdom; School of Psychological Sciences, Birkbeck, University of London, Malet Street, Bloomsbury, London, WC1E 7HX, United Kingdom; Institute of Cognitive Neuroscience, University College London, 17 Queen Square, London, WC1N 3AZ, United Kingdom

**Keywords:** action fluency, conscious intention, EEG, readiness potential, volition

## Abstract

Voluntary actions are often accompanied by a clear, preceding conscious experience of intention. However, the nature of this experience, and the neural mechanisms underlying it, have proved difficult to study scientifically. Many previous studies instructed participants to make simple manual actions, and then report their preceding intention only retrospectively. We combined a typing-based verbal fluency paradigm with pseudorandom probes of conscious experience to address these issues, and used electroencephalography (EEG) to explore neural correlates of prospective intention. Across one behavioral and one EEG experiment, involving 51 participants, we found conscious intention emerged over 1 s before estimated action onset. Further, we found an EEG readiness-potential-like activity that was stronger prior to probes that participants reported as interrupting a conscious intention, compared to other probes. In addition, probes that interrupted conscious intention were found to occur after lateralization of the readiness-potential-like activity to the hemisphere contralateral to the hand that participants reported would type the first letter. Our results provide novel evidence for a prospective experience of conscious intention associated with neural processes that precede voluntary actions.

**Significance Statement**

Many models of voluntary action assume that an experience of conscious intention precedes movement onset, but the mechanisms underlying this experience are highly controversial. By randomly probing experiences of intention during an action generation task involving typing-based verbal fluency, we developed a new method for estimating time of conscious intention, and its neural mechanisms. The method estimates that experiences of prospective intention occur over 1 s before action. Concurrent EEG recordings suggested that the EEG signals classically used as precursor indices of voluntary action are also associated with the experience of prospective intention.

## Introduction

In our daily life, we have the frequent experience of willing and initiating voluntary actions, which we here term “volition.” The nature of volition, however, remains poorly understood, and the very coherence of the concept is controversial (Van Inwagen 1975; Deery 2015). Experimental psychologists have attempted to investigate volition scientifically, but the lack of any consensus definition has limited progress (Haggard 2008; Frith 2013; Kranick and Hallett 2013; Fried et al. 2017; Balleine 2019; Hills 2019). Fried et al. (2017) proposed a cluster definition, involving a set of features that jointly contribute to making an action volitional: (i) the agent is conscious of their action, (ii) the action is innovative or spontaneous, (iii) the action has no immediate external trigger, (iv) the action is goal-directed, or reasons-responsive. Most neuroscientific studies have focussed on just one of these aspects (eg Libet et al. 1983; Maoz et al. 2019), and few studies have addressed them in combination.

The recent experimental study of volition arguably began with the work of Libet et al. (1983). In their experiment, participants were asked to make voluntary hand movements spontaneously when looking at a rotating dot on the screen, while brain activity was recorded by electroencephalography (EEG). After a movement was made, the clock then stopped in a random position, and participants reported the time at which they experienced the intention to act (“the time of appearance of their urge to move” in Libet’s words). The clock provides an estimate of the time of conscious intention (“W time”). W time was approximately 200 ms before action onset, while the beginning of the EEG readiness potential (RP), a common precursor of voluntary action, occurred some 300 ms before that in Libet’s data. Taking the onset of the RP as a marker of the brain initiating the action (Kornhuber and Deecke 1965), Libet argued that these results showed that voluntary actions may be initiated unconsciously.

While Libet’s results opened the path to experimental studies of volition, they also attracted lasting controversy. These discussions generally hinge on methodological difficulties in (i) eliciting truly voluntary behaviors in such experiments, and (ii) measuring the conscious experience relating to these behaviors, and, more recently, (iii) interpreting the relation between action-locked RPs and the conscious initiation of action (Haggard 2024). We now discuss these in order.

In the Libet task, the participant’s action is “meaningless” (Maoz et al. 2019) in the sense that goals and reasons for action remain unclear. Thus, the actions lack some features of everyday volition. Some experiments allowed participants to choose between a few alternative actions, but the effects of manipulating choice set size have been inconsistent (Haggard and Eimer 1999; Takashima et al. 2020; Welniarz et al. 2021). A possible reason could be that these actions are still much constrained by experimental instructions—participants are allowed to choose from a few alternatives, but not from an open-ended choice space. An ideal experimental paradigm for voluntary action might perhaps allow naturalistic and non-instructed voluntary actions chosen from a plausible set of alternatives, with meaningful consequences.

Libet’s experiment used post-action self-report as a measure of conscious experience of intention. Such retrospective reports of the time of conscious intention may be biased by the action itself. These postdictive biases are often thought to reflect processes of perceptual attraction or cue combination. Since the action *follows* the intention, any postdictive bias would then shift the reported time of intention closer to the time of action (Scott 1998; Dominik et al. 2017).

**Figure 1:**
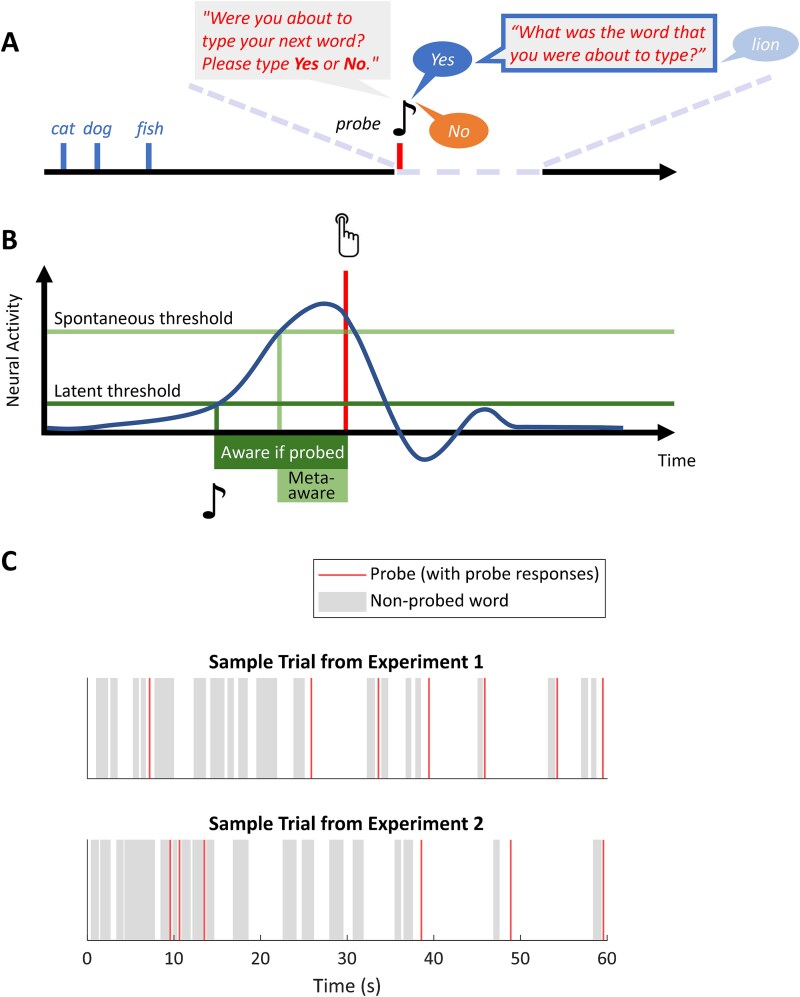
**Experiment logic. (A) Schematic procedure on an illustrative trial.** Participants typed words from a given category (eg animals). During the interval between words, they might hear a random probe tone, which was immediately followed by a question on the screen: “Were you about to type your next word?”. Participants manually responded “Yes” or “No”. If they responded “Yes,” they were then asked “What was the word that you were about to type?”, and entered their response. The period indicated with dashed lines shows the brief pause in the fluency task following each probe, during which participants report their awareness. The generation task resumed after their report. **B. The two-threshold model of intention (adapted from**  Matsuhashi and Hallett (2008)**).** Participants may become aware of their intention to act, even when action-related neural precursor activity is still low, if they are specifically probed (*latent threshold*). At a higher level of neural activity, they become meta-aware, and can spontaneously report their intention. (**C) Sample trials from Expts 1 and 2.** Each gray bar represents the period of typing a word, and the red line represents a probe. Note that the time used to report awareness following a probe (indicated by the dashed line in panel A) is not shown. The trial was “paused” when participants responded to the probe and resumed after they finished reporting their responses to awareness questions.

Matsuhashi and Hallett (2008) proposed a model which recognizes multiple levels of premovement conscious intention. Motor preparation that reaches an early “latent threshold” may be enough for the participant to report a conscious intention if their attention is specifically drawn to it (cf. Parés-Pujolràs et al. 2019). In contrast, the normal experience of intending to initiate an action occurs at a later stage when a higher “spontaneous threshold” is reached (Fig. 1B). When participants retrospectively report the time of their intention or “urge” to move, as in Libet-type experiments, they presumably report the later moment of spontaneous intention. Matsuhashi and Hallett further hypothesized that the “latent threshold” for intention can be sampled by a probe stimulus. As shown in Fig. 1B, the hypothesized thresholds occur in order before action onset. In the very early stage of action preparation, people almost have no access to their conscious intention, even if probed. After the first, latent threshold is crossed, then a probe stimulus drawing a participant’s attention to a current intention might lead to participants acknowledging that they had an intention to act. In contrast, once activation reaches the later spontaneous threshold, people gain momentary awareness of intention without needing to be probed.

The study of volition has historically been linked to measurements of RPs (Kornhuber and Deecke 1965). The RP is calculated by averaging EEG epochs preceding multiple instances of voluntary action, and takes the form of a ramp-like negative-going average potential. However, the relationship between such neural precursors and subjective experience of intention remains controversial (Fried et al. 2011). Several alternative neural signals have been linked to volition. Lateralized readiness potential (LRP), the hemispheric asymmetry of RP associated with hand-specific motor preparation, and beta-band EEG activity are also often identified as markers of different stages of action preparation (Pfurtscheller 1981; Haggard and Eimer 1999; Saleh et al. 2010; Parés-Pujolràs et al. 2023). LRP was argued to be a neural correlate of conscious intention (Haggard and Eimer 1999).

However, recent cognitive theories have questioned the interpretation of the RP as a marker of post-decisional preparation, and instead see it as an artifact of back-averaging stochastic noise in the *input* to a decision process (Schurger et al. 2012). Further, these theories suggest that *all* action-locked measures of volition, not only the classical RP, involve a biased sampling of neural data, that complicates any attempt to identify unique neural correlates of intention (Schurger et al. 2012). In particular, action-locked approaches cannot easily exclude the possibility that a putative neural correlate of intention might also occur at other times unrelated to upcoming action. The pseudorandom probe approach developed by Matsuhashi and Hallett (2008) may avoid some of these sampling biases, as the neural activity is not sampled only around the time of action.

Here we provide a novel experimental approach to volition by using typing-based verbal fluency as a context for eliciting voluntary actions, and by using probe sampling methods to probe intentions before action. The method addresses methodological difficulties of previous studies by (i) eliciting goal-directed and relatively unconstrained voluntary actions, (ii) obtaining prospective and ecologically-valid measures of conscious intention, as well as (iii) providing unbiased or less-biased evidence to clarify the relationship between conscious intention and the neural precursors of action. Verbal fluency tasks require participants to generate as many words as possible in a certain category within a given period of time (Fogel 1962; Butters et al. 1987). They are often used as measures of executive function for clinical assessments (Laurent et al. 2000; Jones et al. 2006; Gustavson et al. 2019). Appropriate words must be retrieved from verbal long-term memory, and transformed into actions. We asked participants to **type** the words they generated, so the actions are manual keypresses, rather than speech. Thus, while verbal fluency has generally been used to study semantic and lexical processing, it also meets the key criteria for volitional action mentioned above.

Typing verbal fluency clearly involves a sequence of cognitive processes, such as memory search, and then successful word retrieval, followed by action preparation and finally manual typing actions. We inserted probe signals pseudorandomly into this processing stream. When the participant’s performance was interrupted by the probe signal, we asked whether they were about to type a word or not, and (if so) which word it was (see Fig. 1A for trial procedure and Fig. 1C for sample trial behavior). A distinct advantage of our paradigm is that this question is straightforward and relatively easy for participants to answer, in contrast to previous methods of reporting conscious intention, such as identifying the moment of first feeling an “urge to move” (Libet et al. 1983).

To summarize, the combination of typing-based verbal fluency combined with pseudorandom probes meets several criteria for volition: (i) participants have clear and accessible conscious intention to their action, (ii) the action is spontaneous and innovative as participants freely generate words from a large semantic space, (iii) the action has no immediate external trigger, being driven by participants’ search in long-term memory, and (iv) the action is goal-directed and reasons-responsive, as the fluency task gives participants a clear goal and meaningful context for action. We present here two experiments using this method to investigate the temporal relationship between conscious intention and the upcoming action (Expts 1 & 2), and the relationship between conscious intention and the EEG precursors of voluntary action (Expt. 2).

## Materials and methods

### Participants

Based on the sample size suggested in a paper using a similar language task (Brysbaert and Stevens 2018), we chose a minimum sample size of 20 participants. Pre-registrations were deposited at https://aspredicted.org/, with reference numbers 126028 and 144817. Experiments 1 and 2, were completed by 24 and 29 participants recruited online through the UCL Psychology Subject Pool. All participants were native English speakers and could touch-type above 35 words per minute, as confirmed by an initial screening test, using standard finger-key assignments, and without looking at the keyboard. Participants were paid with either credits (1 credit/h) or cash (£9.2/h and bonus). The study was approved by the UCL Research Ethics Committee, and followed the principles of the Declaration of Helsinki. The Approval ID Number is ICN-PH-PWB-22-11-18A. All participants signed the consent form before taking part in our experiments.

One participant was excluded from Experiment 1 for low rates of word generation (<10 words/minute). In the end, 23 participants were included in the analysis of Experiment 1 (3 males), aged between 18 and 36 (mean (*M*) = 23.61, standard deviation (*SD*) = 4.57). One participant was left-handed, one ambidextrous, others were right-handed. For Experiment 2, one participant was excluded for behavioral analysis because of a technical error, resulting in a total of 28 participants (8 males) aged between 18 and 40 (*M* = 23.68, *SD* = 6.04). In the EEG analysis, 2 more participants were excluded due to technical difficulties with EEG signals, leaving 26 participants in the final analysis (8 males), aged between 18 and 36 (*M* = 23.92, *SD* = 6.18). 3 participants were left-handed, others were right-handed. Handedness information was obtained through self-report. Note that lateralization analysis (see later) was determined by the hand used to type the first letter of a word, rather than by dominance.

### Set-up

Experiment 1 was developed using MATLAB R2022b and Psychtoolbox-3.0.18 (Brainard and Vision 1997; Pelli 1997; Kleiner et al. 2007). Participants were seated approximately 60 cm away from a 14-inch screen.

Experiment 2 was developed using MATLAB R2015b and Psychtoolbox-3.0.11 (Brainard and Vision 1997; Pelli 1997; Kleiner et al. 2007). Participants were seated approximately 60 cm away from a 20-inch monitor. EEG signals were recorded using a 32 channels EEG cap (g.GAMMAcap) in 26 scalp sites (Fz, FCz, Cz, CPz, Pz, POz, FC1, FC2, C1, C2, CP1, CP2, F3, F4, C3, C4, P3, P4, O1, O2, FC5, FC6, CP5, CP6, F7, F8) with active electrodes. The cap was connected to the g.Recorder v4.12.03 using g.GAMMAbox and g.USBamp (g.tec medical engineering GmbH, Austria). The sampling frequency was 256 Hz, and high-pass and low-pass infinite impulse response Butterworth filter was applied, with 0.01 Hz and 100 Hz frequency respectively. All EEG electrodes were online referenced to the left ear lobe. EOG electrodes were online referenced bipolarly to each other. Vertical electroocular activity was recorded from electrodes above and below the left eye and horizontal electroocular activity the temples outside both eyes.

### Task

A typing verbal fluency task was used. 24 categories were presented to participants, in a different random order for each participant (see *Supplementary Material*). Participants had to type as many words as possible from each category, for a period of 60 s. Participants could take as long a break as they wished between each of the 24 tests.

Participants saw on a gray background a small white rectangle where they would type in the words they generated. Participants pressed the *Enter* key at the end of each word. The word would then disappear, ready for them to generate a further word.

### Time of awareness of intention to act report

We probed participants with a tone during the interval after typing one word, but before typing the next (see Fig. 1A). The probe was a beep sound (1000 Hz, 50 ms) accompanied by the question appearing on the screen: “Were you about to type your next word?” Participants could answer “Yes” or “No” to this question. If they answered “Yes,” a further question would appear: “What was the word that you were about to type?” They would then type the word. They were also allowed to type “I don’t know”/“idk”/“I forgot.” The timing of the probe was drawn from a uniform random distribution limited by the end of the previous word and the estimated time of the next action. The estimated time of the next action was based on an average of previous inter-word intervals in the current category. The probability of the probe happening in any given inter-word interval was set to 0.3 for the first half of each 60 s trial, and 0.7 for the second half, based on published observations (Ovando-Tellez et al. 2022; Bieth et al. 2024) and our own pilot study. These show rapid word generation initially, followed by effortful search and less frequent word generation later in the trial. This later phase was of more interest in the current study, since it has been specifically associated with de novo generation as an executive function (Ovando-Tellez et al. 2022; Bieth et al. 2024). The actual probabilities achieved were limited by the rule that consecutive inter-action intervals should not both be probed. Thus, the actual probabilities of a given interval being probed were 0.23 and 0.41 on average for the first and second halves of each trial respectively.

### Procedure

Participants sat at a computer in a quiet room. They performed a pre-test of touch-typing speed (https://www.typingtest.com/). If they passed the criterion of 35 words per minute, they would enter the main experiment. For Experiment 2, participants remained seated while EEG equipment was being set up before the practice. Their skin was cleaned, electrodes attached to their scalp with gel facilitating the connection, signal quality checked for formal recording.

Initial practice was the same as the formal experiment, except that it did not have probes in the trial. It consisted of one category only (“Animals”). Participants were instructed to type the names of as many items as possible belonging to the category, within 1 min, while avoiding proper names. They then began the experimental task. They first received instructions on the main experiment, and particularly on probe stimuli. They were instructed to respond to the question accompanying probe sounds (“Were you about to type your next word?”) by typing “Yes” or “No” accordingly. The details of the task can be found in the previous two sections.

Participants’ typing production was checked manually. Words not belonging to the required category were excluded. Minor mis-spellings of words were accepted as valid responses, but unintelligible words were excluded. Words that were generated after a pause of more than 15 s were treated as outliers to avoid distorting attempts to estimate the onset of the next word. An average across participants of 7.3% and 2.2% of the words generated were thus excluded for Expts 1 and 2 respectively.

For probe responses, two classes of abnormal responses were identified: finger error data (purple in Fig. S7B) and unclassified data (green in Fig. S7B). Finger error probe responses are defined as responses where the participant was assumed to have intended to type “Yes” or “No,” in response to the question immediately following each probe “Were you about to type your next word?”. We defined finger error responses as typing a strong beginning with “T” or “U” (errors when the “Y” of “Yes” was presumably intended), and “B” or “M” (errors when the “N” of “No” was presumably intended). Participants usually corrected these finger errors later. In Expt 2, 1.6% of the probe responses contained finger errors (purple in Fig. S7B, right). Finger errors were reclassified as “Yes” and “No” responses, and were used in later analyses. In Expt 1, we only recorded those keypresses that were not subsequently deleted by participants, so finger errors could not be identified and classified.

A further subset of typing responses after the probe could not be classified as “Yes” or “No” responses to the “Were you about to type your next word?” question. These were labeled as unclassified and excluded from all analyses. They formed 0.8% and 2.4% of responses to the post-probe question in Expts 1 and 2 respectively. From Expt 2 (where all typing keypresses were recorded even when deleted later), we could speculate that these unclassified responses were often words generated from the verbal fluency task, rather than “Yes/No” responses to the post-probe question. Participants may have been unable to cut-off the stream of voluntary action generation when they heard the probe. Their action was too close to the probe onset and had passed the so-called “point of no return” (de Jong et al. 1990; Matsuhashi and Hallett 2008; Logan 2015). By inspecting the distribution of response times to the post-probe question in Expt 2 (see Fig. S7B, right), we noted that these keypresses often occur very shortly after the probe. From inspecting the distribution, we could estimate that the “point of no return” in our experiment may be around 600–800 ms.

### Chronometric modeling

The data was then analyzed using RStudio (R version 4.2.2) (R Core Team 2022). We used a 3-step modeling pipeline to (i) predict the likely time of an upcoming action, (ii) characterize the time of each individual probe relative to the estimated time of the upcoming action, and (iii) consider participant’s awareness of intention relative to (ii). We describe each step in detail below.

#### Step 1—use inter-word interval model to describe the pattern of action onsets

An exponential mixed-effects regression was fitted to inter-word intervals (IWIs) from non-probed words, with observation time within the trial as predictor and with random intercept and slope. This captures the well-documented increase in IWI with time, as verbal fluency decays. The model can be used to estimate when an action is likely to occur when it is interrupted by a probe.


$$ \mathit{\ln}\left({IWI}_{ij}+1\right)=\left({b}_0+{s}_{0j}\right)+\left({b}_1+{s}_{1j}\right){T}_{ij}+{\varepsilon}_{ij} $$

where.

*i* = each observation,

*j* = each participant,

*IWI* = inter-word interval,

*T* = time of each observation in the trial (0–60s),

*b*_0_ and *b*_1_ are intercept and slope for fixed effect, while *s*_0*j*_ and *s*_1*j*_ are participant-specific intercept and slope to capture each participant’s individual behavior.

#### Step 2—calculate latency of each probe relative to estimated time of impending action

For each probe event, the model fitted at step 1 was used to predict the IWI for the upcoming word that would have occurred at that point in the trial, given this participant’s baseline IWI and increase of IWI over time. This yields an **estimated** onset time for the word that was interrupted. As the onset of the next word must be estimated, the data value for *T* was the time of the probe in the trial (0 to 60 s), instead of the upcoming next word in the trial. The time of the probe relative to this *estimate* of likely word onset was then calculated.

#### Step 3—relate awareness of intention to probe latency

A mixed-effects logistic regression was used to predict the awareness report (Yes/No) from the probe time relative to estimated onset Participants were modeled with a random intercept and random slope, meaning that the function was allowed to vary across individuals. The 50% crossing point of this function, the time prior to estimated action at which participants became aware of the intention to act, is taken as W^*^.


$$ logit\left(p\left({A}_{ij}=1|{R}_{ij}\right)\right)=\left({b}_0+{s}_{0j}\right)+\left({b}_1+{s}_{1j}\right){R}_{ij}+{\varepsilon}_{ij} $$

where.

*i* = each observation,

*j* = each participant,

*A* = awareness,

*R* = relative time to the next action,

*b*_0_ and *b*_1_ are intercept and slope for fixed effect, while *s*_0*j*_ and *s*_1*j*_ are participant-specific intercept and slope to capture each participant’s individual behavior.

Finally, this 3-step pipeline was run twice, in two parallel analyses, using participant as a random factor, and using category as a random factor, respectively.

### Electroencephalography (E‌EG) preprocessing

The EEG analysis was done using EEGLAB v2023.0 (Delorme and Makeig 2004). The preprocessing was done through the following steps:

(1) Re-referencing

The average of the two mastoid channels was used for re-referencing the recorded EEG (A1 and A2, also named as M1 and M2 in some conventions). In a few cases, one mastoid signal was found to be noisy and of poor quality, so the other was used. 5 participants had bad A1 signal, so A2 was used; 1 participant had bad A2 signal, so A1 was used.

(2) Filtering

A band-pass filter of 0.1 Hz to 30 Hz was applied to the data. This range could keep the potential slow precursor signals of interest for voluntary action, while removing most of the noise.

(3) Downsampling

The original 256 Hz recording was downsampled to 200 Hz for computational convenience.

(4) Epoching

An epoch of −1 to 0.2 s relative to the onset of probes was used for potential data. To avoid edge effects, analysis of spectral data was restricted to a narrower window of −800 to 0 ms.

(5) Baseline correction

Baseline correction was performed using the amplitude values −10 to 0 ms prior to each probe. This baseline was carefully chosen for consistency with previous RP studies (Khalighinejad et al. 2018), and to avoid having to pick premovement baseline periods on the basis of unwarranted assumptions about when precursor neural activity begins.

(6) Eye movement removal with ICA

Independent component analysis (ICA; EEGLAB runica algorithm) was used to remove eye movement artifacts. Vertical eye movements were identified by their characteristic frontal topography (focal frontopolar positivity) and stereotyped time courses in vEOG channels (sharp deflections). Horizontal eye movement components were identified by their lateralised frontal topography (bilateral opposing polarity) and stereotyped time courses in hEOG channels (sustained step-like shifts). Removed components and post-ICA signals were inspected to confirm that eye-movement-related EEG components had been removed. Across participants, 4.77 components on average were removed (*SD* = 3.31).

(7) Epoch boundary rejection

Epochs with signals exceeding +/− 120 μV in channels of interest (FCz, C3, and C4) were excluded to avoid possible artifacts (Parés-Pujolràs et al. 2019). 2.9% of epochs were excluded, resulting in an average of 92 valid epochs per participant.

### E‌EG measures

Our EEG analyses were inspired by three neural measures that have been associated with impending voluntary action: RP, beta-band EEG, and lateralized RP (LRP). These measures are conventionally computed in action-locked analyses as markers of precursor activity such as action preparation (Pfurtscheller 1981; Haggard and Eimer 1999; Saleh et al. 2010; Parés-Pujolràs et al. 2023). Here, however, we have locked the EEG analysis to the *onset time of the probe signal*.

For RP and beta-band EEG measures of neural preparation, we could test whether the corresponding neural precursor pattern is *stronger* prior to probes that happen to interrupt a conscious intention to act, compared to those that do not. The clear direction in our prediction justified the one-tailed test used in our analyses. Such findings would suggest that an experience of conscious intention is *prospectively* present as a readout of ongoing neural preparation for action, rather than being a retrospective confabulation triggered by the fact of having acted, in which people construct a post-hoc narrative of having intended, to form a consistent experience with their action (Dennett 1993; Wegner 2004). We could therefore speak of a *non-inferential* subjective experience associated with neural preparation for action.

For EEG amplitudes, we assumed that a readiness-potential-like signal might be present prior to the probe. However, this would have a smeared and desynchronized form since the RP is considered as linked to the impending action, while probe timing is not systematically locked to action onset. Our core research question was whether this RP-like signal was associated with a conscious intention of the impending action. This analysis focussed on electrode FCz, a key site for the classical RP (Khalighinejad et al. 2018; Takashima et al. 2020; Travers et al. 2020). Since our baseline period was taken immediately before the probe (see *Methods*), an RP-like component would correspond to a positive potential with a negative-going trend in the pre-probe period.

The beta-band EEG activity at FCz was measured by a seven-cycle continuous Morlet wavelet transform in the frequency range of 13 to 30 Hz (Parés-Pujolràs et al. 2023). The beta-band amplitude was calculated as the square root of the beta band power. The time window chosen was from −800 ms to the time of the probe: earlier timepoints showed epoch-edge effects from discrete time-frequency transformation, as applying the wavelet close to the epoch boundary would produce unreliable estimates.

The calculations for lateralized activity used response from participants when they reported awareness of conscious intention for the upcoming word. When participants responded upon probing that they were about to type a word, their answer to the following question “What was the word that you were about to type?” can indicate *which hand* would have been used to initiate the interrupted action. This is because touch-typing conventions strictly assign some letters to the left hand and others to the right hand. This allowed us to calculate a probe-locked analogue of the conventional LRP, obtained by the double subtraction method: (C3-C4)L-(C3-C4)R (Eimer 1998). The LRP measure captures an increase in cortical negativity contralateral to the action, beginning after the classical RP onset, and typically some −500 to −200 ms prior to movement (Deecke et al. 1976; Haggard and Eimer 1999; Shang et al. 2016). In our probe-locked data, double subtraction produced an *LRP-like* potential, analogous to the probe-locked *RP-like* potential discussed above. The LRP-like potential could only be defined for probes associated with conscious intention, as participants only reported the word they were going to type, and thus the predicted lateralization of the first letter, if they reported a conscious intention when probed. Therefore, the research question becomes whether an LRP-like potential is or is not present prior to a conscious intention. We have made a directional prediction regarding the LRP, which we explain in the results section.

## Results

In two experiments, we combined typing-based verbal fluency tasks with probe sampling in order to study volition. Experiment 1 investigated the timing of conscious intention using these methods. Experiment 2 replicated the design, and additionally measured the EEG correlates of intention.

We first provide a behavioral overview of our data (see Table 1). Participants generated 12.60 (±.36) and 14.04 (±.46) words per minute in Expts 1 and 2, respectively (values are *M* ± standard error of the mean (*SEM*), except for probe counts which are *M* ± *SD*). Their inter-word intervals were 2773 (±153) ms and 2163 (±89) ms in Expts 1 and 2, respectively. The distribution of inter-word intervals preceding normal action is shown in Fig. S7A*.* The probe timings relative to the preceding word were 678 (±35) ms and 620 (±32) ms in Expts 1 and 2, respectively. The number of probe responses in total was 89.04 (±13.39) and the numbers for aware and unaware responses were 40.13 (±11.28) and 48.91 (±16.18) in Expt 1. The total number was 91.04 (±16.49), and 45.46 (±15.67) and 45.57 (±17.47) for aware and unaware responses in Expt 2. The distributions of probe timing across the 60-second trial for sample participants and all participants in Expts 1 and 2 are shown in Fig. S8A and B.

**Table 1:** Descriptive statistics for key measures across experiments.

	**Wpm**	**IWI (ms)**	**Probe time to last word (ms)**	**Probe count**		
				**Total**	**Aware**	**Unaware**
**Expt 1**	12.60 (.36)	2773 (153)	678 (35)	89.04 (13.39)	40.13 (11.28)	48.91 (16.18)
**Expt 2**	14.04 (.46)	2163 (89)	620 (32)	91.04 (16.49)	45.46 (15.67)	45.57 (17.47)

*Note:* values are displayed as *M* (*SEM*), except for Probe count values which are *M* (*SD*).

### Conscious experience of intention and action (experiment 1)

The typing-based verbal fluency task was used as a context to elicit voluntary actions, while probe signals were used to obtain reports of conscious intention to act (for trial behavior, see Fig. 1C). The probes were timed to occur pseudorandomly in the interval between the end of typing one word and the start of the next (for details, see *Methods*). We predicted that probing participants closer to the expected time of their typing the next word, should make them more likely to report an intention (ie being about to type the next word), compared to probing them earlier.

**Figure 2:**
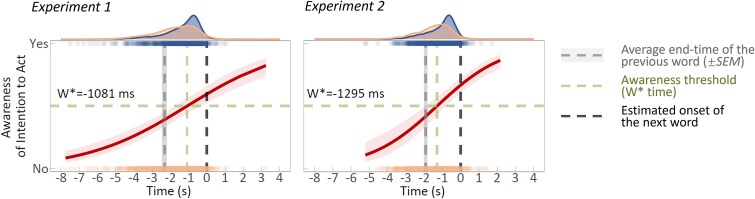
**Time of intention awareness in experiments 1 (*n* = 23) and 2 (*n* = 28).** Time 0 is the ***estimated*** onset of the next word. Each datapoint represents a single probe, eliciting a response indicating awareness of intention to type the next word (dark blue), or absence of intention (light orange) when probed. Mixed-model logistic regression fits are shown—the 50% cut-off of these fits may be used to estimate the time of conscious intention (W^*^). Marginal density functions are shown above each plot. Note the separation of “Yes” from “No” responses in the distributions, beginning around W^*^ time.

Figure 2 (left) shows the results from behavioral Experiment 1. Data are aligned to the expected onset of the word interrupted by the probe (see *Chronometric modeling* section in *Methods* for the estimation procedure). A logistic function has been fitted to the group data, using participants as a random factor. The monotonic curve shows that the probability of reporting an intention when probed increased as the expected onset of the next action approached. We use the term W^*^ to refer to the time of awareness of the intention to act calculated from the 0.5 probability threshold of this curve. The random-slopes model computed a W^*^ time of −1081 ms (95% CI: [−1766, −395]). A similar trend was shown with individual model fits from each participant’s probe data (Fig. S1A). The average W^*^ time was −1023 ms (*SEM* across participants = 304 ms). The distribution of W^*^ time shows that most participants had their awareness of intention between −2000 and −1000 ms relative to the action (Fig. S1B). For model fit diagnostics, please see Table S1.

Re-analyzing the data with verbal fluency category (rather than participant) as a random factor produced similar conclusions, though with slightly different estimates (Fig. S2). The second-level modeling revealed an increase of the awareness drawing near to the expected onset of the next action, with a general W^*^ time of −954 ms (95% CI: [−1452, −456]). ​When averaged from individual-category fits, the average W^*^ time was −682 ms (*SEM* across categories = 264 ms). This difference results from the differential weightings given to different categories in the mixed model.

### Neural correlates of conscious experience of intention to act (experiment 2)

Experiment 2 broadly replicated the psychophysical data of Experiment 1 in a new sample of participants (Fig. 2). Again, the positive slope of the curve showed that the probability of reporting an intention when probed increased as the expected onset of the next action approached. The computed W^*^ time was −1295 ms (95% CI: [−1819, −771]). The same trend was shown in individual fits (Fig. S3), which had an average W^*^ time of −1131 ms (*SEM* across participants = 137 ms). When category rather than participant was used as a random factor (Fig. S4), the second-level model obtained a W^*^ time of −1199 ms (95% CI: [−1399, −998]), while results from individual categories had an average of −1171 ms (*SEM* across categories = 58 ms).

In addition to behavioral data, Experiment 2 recorded EEG, and investigated potential neural correlates of the intention to act. In contrast to conventional RP analyses of intention, we have locked our main EEG analysis to the *onset time of the probe signal* (for details, see *Methods*), and considered what happens prior to the probe.

**Figure 3:**
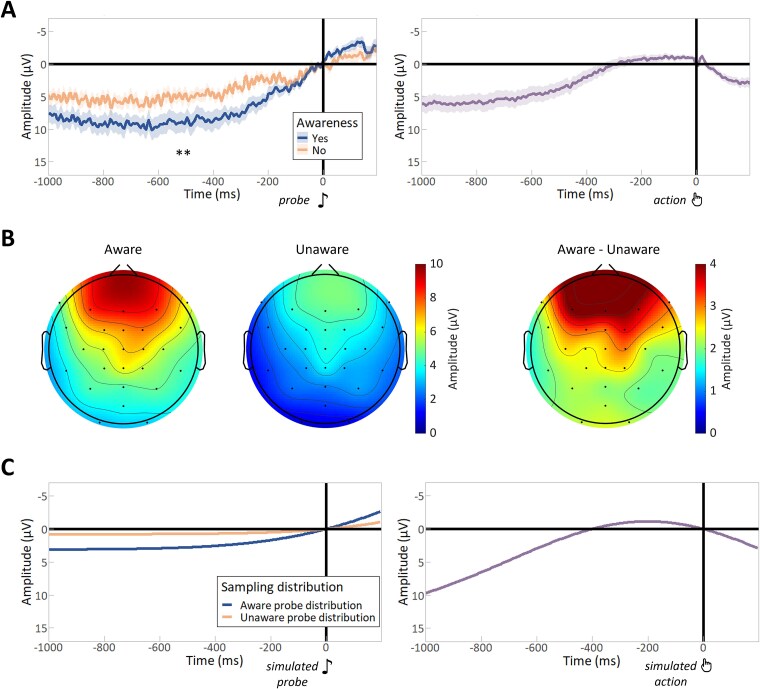
**EEG amplitudes at electrode FCz related to intention (*n* = 26). (A) EEG amplitude (±*SEM*) around the time of the probe (left) and action (right).** Mean EEG amplitude from −1000 to 0 ms relative to probe onset showed a stronger negative-going, readiness-potential-like deflection when participants were aware of their intention to act (dark blue trace) than when they were unaware (light orange trace). The form of this readiness potential-like pre-probe activity resembles a desynchronized version of the classic readiness potential shape observed during the same time window prior to action onset. ^**^ indicates a significance level of *P* < 0.01. (**B) Topographic plots of average EEG amplitude before probe onset in aware and unaware conditions (left) and their difference (right).** The plot shows average EEG amplitude interpolated across the scalp in aware, unaware conditions, and the difference between them, averaged across a time window from −1000 to 0 ms relative to probe onset. (**C) Simulation of the effects of desynchronization on average RP (left) compared to classical RP (right).** Synthetic RP signals (right) were generated using a Gaussian function as a convenient proxy function with a form mimicking the observed form of action-locked RP, and we then desynchronized 100 instances of this function, and averaged the result (left). For the desynchronization simulation, we used the mean and standard deviation of our data (aware distribution: μ = −1096 ms, σ = 53 ms, for unaware distribution: μ = −1435 ms, σ = 65 ms). Individual trial RPs were re-aligned before averaging to random time points drawn from these normal distributions, rather than to the action itself. This simulates the effects of randomly probing prior to action. Note the loss of temporal detail and reduction of amplitude in desynchronized simulations relative to action-locked simulations, and the difference in amplitudes between simulated RP signals from aware or unaware distributions.

After pre-processing (see *Methods*), EEG amplitudes were compared prior to probe signals that occurred when participants were or were not aware of the intention to type a next word. We predicted a *stronger* negative-going amplitude prior to probes that happen to interrupt a conscious intention to act, compared to those that do not. We indeed observed such an RP-like component (Fig. 3A), and found that mean EEG amplitude during the 1 s before the probe was greater when participants were aware of their intention than when they were unaware (*t*(25) = 2.72, *P* = 0.006, Cohen’s *d* = 0.53, one-tailed). The topography of the RP-like potential (Fig. 3B) *shows a clear frontal focus* when participants were aware of their intention. This resembled the topography prior to typing the initial letter of each un-interrupted word (Fig. S5A), suggesting that the probe-locked and action-locked activity might represent different temporal samplings of the same underlying neural activity. Further, we compared the form of the RP-like potential first with the form of uninterrupted action-locked RPs (Fig. 3A, right) and, second, with simulations of the effects of desynchronising an illustrative action-locked RP signal (Fig. 3C). These comparisons suggested that the RP-like potential resembled a smeared, desynchronized version of the classical action-locked RP. Thus, when participants reported that a probe had interrupted their conscious intention prior to action, then the cognitive processing associated with classical RP was already present.

**Figure 4:**
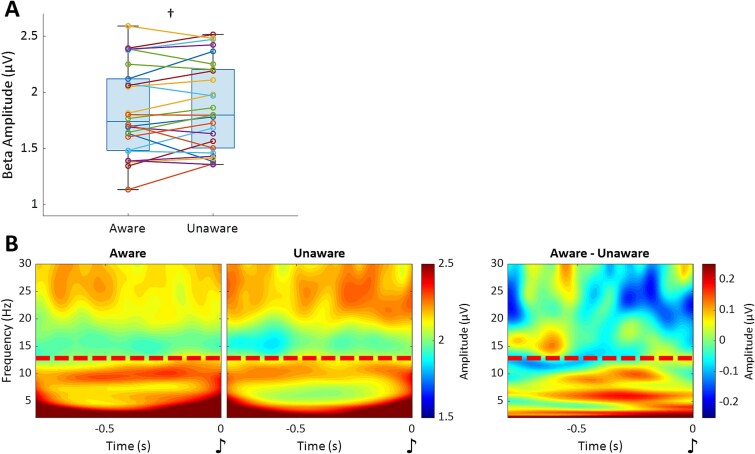
**EEG beta-band amplitudes (13 to 30 Hz) at electrode FCz before probe onset (*n* = 26). (A) Mean beta-band amplitude.** The plot shows mean beta amplitude in aware and unaware conditions averaged across a time window from −800 to 0 ms relative to probe onset. † indicates a trend significance level of 0.05 < *P* < 0.10. **(B) Grand-average EEG beta-band amplitude before probe onset in aware and unaware conditions (left) and their difference (right).** The plot shows beta amplitude in aware, unaware conditions and their difference in a time window from −800 to 0 ms relative to probe onset.

Beta-band amplitude was predicted to have *a greater decrease* prior to probes that happen to interrupt a conscious intention to act, compared to those that do not. A paired *t-*test showed a marginally lower mean beta amplitude prior to probes associated with conscious intention than to probes not associated with conscious intention (*t*(25) = −1.58, *P* = 0.06, Cohen’s *d* = −0.31, one-tailed), though this did not reach the conventional boundary of significance (Fig. 4).

**Figure 5:**
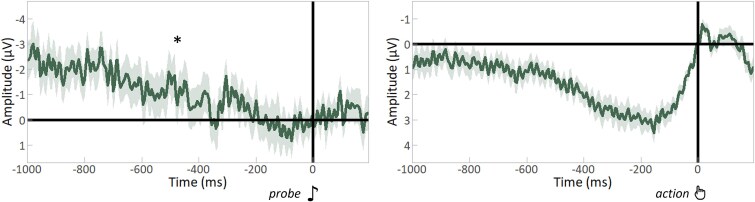
**Amplitude of an LRP-like potential related to prospective intention (*n* = 26).** LRP-like amplitude (±*SEM*) locked to the time of probes associated with conscious intention (left) and locked to the time of action (right). LRP-like amplitudes were calculated for epochs with probes that elicited reports of conscious intention by double subtraction method (Eimer 1998) at electrodes C3 and C4, from −1000 to 0 ms relative to probe onset. The probe-locked LRP-like potential resembles a desynchronized version of the classical action-locked LRP. ^*^ indicates a significance level of *P* < 0.05, one-tailed (see text for explanation).

Classical action-locked LRPs calculated by double-subtraction method typically have a positive peak just before action onset (Haggard and Eimer 1999, see Fig. S6, white arrow). Our *probe-locked* LRP-like potential could, however, have either positive or a negative sign, according to when the probe occurred relative to the divergence between contralateral and ipsilateral cortical activity that precedes voluntary action (see Fig. S6, column 4). If probes occurred before any lateralization of preparatory activity, then probe-locked LRP would be flat prior to the probe (Fig. S6, blue arrow). However, we could predict that our probes would often occur during the rising portion of the action-locked LRP for three reasons: (i) our probes were designed to interrupt each participant’s action preparation, (ii) the peak of the uninterrupted action-locked LRP was around 200 ms before action (Fig. 5, right), and (iii) we excluded probes that occurred after a “point of no return” estimated at some 600 to 800 ms prior to action (see *Methods* and Fig. S7B, right). This means that the remaining, included probes probably occurred more than 600 ms prior to the likely time of action. Given these constraints, we could predict that included probe-locked LRPs would be negative if the probes had occurred after divergence between contralateral and ipsilateral cortical preparatory activity (row 3 in Fig. S6, red arrow), but close to 0 if the probe occurred before divergence (row 4 in Fig. S6, blue arrow).

We found the mean probe-locked LRP-like potential amplitude to be significantly less than 0 during the 1 s prior to probes associated with conscious awareness of intention (*t*(25) = −2.04, *P* = 0.03, Cohen’s *d* = −0.40, one-tailed) (Fig. 5, left). A one-tailed test is appropriate because we hypothesized that the probe-locked LRP would be negative (red arrow) or flat (blue arrow). The LRP-like potential prior to probes thus looks like a smeared, desynchronized version of the classical LRP (Fig. 5, right). Thus, some lateralized activity was found prior to those probes for which participants reported being about to type the next word when probed. To address concerns that single-mastoid referencing may introduce a lateralization bias, we reanalyzed LRP results for the 20 participants using an average reference. The pattern of results was consistent with the full group analysis, though the effect reached only marginal significance in this subset (*t*(19) = −1.48, *P* = 0.08, Cohen’s *d* = −0.33, one-tailed), likely reflecting reduced statistical power in the smaller subset (Fig. S10). We also computed lateralization for beta-band activity, but found non-significant results (for words beginning with left hand: *t*(25) = .41, *P* = 0.66, right hand: *t*(25) = −.32, *P* = 0.37, one-tailed tests; see Fig. S11). Taken overall, we found modest evidence for presence of LRP-like activity prior to probe associated with conscious intention. This is consistent with the view that conscious awareness of intention occurs after the onset of lateralized neural preparation.

## Discussion

### Validation of a new method for sampling conscious intention to act

Across two experiments, we have used verbal fluency as an experimental paradigm for studying volition and intention. Experiment 1 showed that an intention to act could reliably be investigated by interrupting participants during a typing-based verbal fluency task, and asking them whether they were about to type the next word in the fluency test. A key advantage of this method is the simplicity and familiarity of the questions it uses to study conscious intention. Participants readily understood the question, could report whether they had an intention for a word when probed. They did not spontaneously indicate any uncertainty about the conscious experience they were asked to detect and describe. This contrasts with the rather unusual request to report “when you first felt the urge to move” used in other studies (Libet et al. 1983). Further, we used a simple statistical model of each participant’s inter-word intervals to place a few such probes pseudorandomly into a 1-min verbal fluency task, and to estimate the interval between each probe and the *likely* onset time of the next word. Thus, while our probes were not fully random, they may avoid some of the biases associated with sampling intentions retrospectively, only after an action has occurred (Schurger et al. 2012).

The probe therefore interrupted the normal processes of fluent action. We asked participants to report whether they felt they were about to type their next word at the time of the probe. A “Yes” response would indicate that the participant had an experience of *prospective intention*, ie they experienced being about to execute a specific, endogenous, task-appropriate action. We used this report in two different types of analysis. First, we could use the reports for mental chronometry (Libet et al. 1983; Matsuhashi and Hallett 2008) to measure how long before the estimated time of action the experience of prospective intention might occur. Our main interest, however, lay in identifying the neural correlates of prospective intention.

The pseudorandom probing method aims primarily to *detect* prospective intentions. However, by representing awareness reports as function of the likely time remaining prior to action we observed that conscious intention grew increasingly likely as time passed, and as the estimated onset of the next action approached. Logistic regression could fit individual models for each participant or each fluency category to describe the temporal evolution of prospective intentions.

The intercept of our logistic regressions suggested that conscious intention appeared over −1000 ms prior to the estimated onset of the next action. Model fit diagnostics suggested that the regression relations were reliable (see *Supplementary Material*). However, as the exponential form of our IWI model is only an approximation of the more complex process, we further explored the possible bias of these estimates by simulation (see *Supplementary Material*). After bias adjustment, our average times of prospective intention (−1881 ms in Experiment 1 and −1791 ms in Experiment 2) were closer to the −1420 ms estimate of latent intention reported by Matsuhashi and Hallett (2008) in their random-probing experiment, than to timings typically reported using retrospective methods (eg −204 ms in Libet et al. (1983); −354 ms in Haggard and Eimer (1999)). This substantial difference between typical prospective and retrospective reports may reflect the low threshold for detecting intentions when probed, compared to spontaneously (Matsuhashi and Hallett 2008), or may reflect a postdictive, delaying effect of action itself on awareness of intention in classical retrospective reports of conscious intention. Taken overall, the psychophysical results are consistent with a precursor experience linked to the cognitive processes of generating voluntary actions. However, our key finding lies in the neural correlates associated with such conscious intention.

In Experiment 2, we investigated three possible neural correlates of prospective intention, focussing on biomarkers reported previously: readiness potential (RP) (Pfurtscheller 1981; Parés-Pujolràs et al. 2023), beta-band oscillations (Parés-Pujolràs et al. 2023) and LRP (Haggard and Eimer 1999). By comparing the neural activities prior to probes eliciting aware vs unaware reports, we aimed to identify possible neural correlates of prospective intention (Parés-Pujolràs et al. 2019). Briefly, we found that a negative-going RP-like signal was present prior to pseudorandom probes designed to interrupt action preparation, and was stronger for probes associated with conscious intention compared to those that were not. On top of that, LRP-like activity had already begun before those probes associated with conscious intention. That is, we found divergence between potentials recorded over the contralateral vs ipsilateral cortex, defined according to the hand that would initiate action as implied by participants subsequent report of the word they were planning to type. The absence of significant beta lateralization may reflect the limited temporal precision of oscillatory measures compared to the LRP. Typing actions are bimanual. Our participants were selected for rapid touch-typing ability. The rapid succession of keystrokes across the two hands may limit the ability to resolve lateralization of oscillatory activities.

The presence of LRP-like probe-locked component sensitive to awareness suggests conscious experience of prospective intention might be linked to the brain generating a *specific* action plan, ie whether the first letter of the impending word will be typed with the left or the right hand. Previous studies using retrospective reports had suggested the LRP could be a neural correlate of conscious intention (Haggard and Eimer 1999; but see also Schlegel et al. 2013), and our data is consistent with this view. We acknowledge that the LRP-like patterns in our dataset constitute only modest evidence, and were subject to some technical constraints regarding EEG referencing. Therefore, these results should be interpreted with caution and would particularly benefit from replication.

We noted a numerical decrease in EEG beta-band amplitudes prior to probes that were associated with conscious intention compared to those that were not. However, this did not reach the conventional level of statistical significance when averaged across the broad time-window that we analyzed. Inspection of the beta-band time course reveals a direction of change consistent with our hypotheses. That is, we saw a shift from higher to lower beta amplitudes in the few hundred ms before onset of the probes associated with conscious intention (Fig. 4B, left), while probes not associated with conscious intention showed changes in the opposite direction. Our analyses have been based on EEG activity within a broad time-window, rather than on identifying the precise time of any given EEG change. We chose a broad time-window approach because pseudo-random probes are inevitably desynchronised relative to underlying neural events. This desynchronization is expected to adversely affect signal-to-noise ratios. Future studies attempting time-resolved analyses would benefit from larger datasets.

### Interpreting inconsistent features of RP-like EEG activity prior to probes

Some aspects of our data are difficult to explain based on our simple working assumption that probes randomly interrupt processes that precede normal voluntary actions. For example, the RP-like potential before probes was greater in amplitude than the true action-locked RP prior to normal actions that were not probed (Fig. 3A). If probe-locking simply extracted a partly-desynchronised subsection of normal action-locked activity, then probe-locked RP-like signals should be *smaller* than classical RPs (Fig. 3C). The fluctuation of task performance over the course of the 60 s fluency trial might partly explain this. However, additional analyses (see *Time course factors* in *Supplementary Material*) showed that the contrasting distributions of probes and word-initiating actions across the 60 s of each trial is not in itself associated with particular differences in neural activity. Instead, sampling bias offers one possible explanation. Our probe-generating algorithm was more likely to interrupt longer rather than shorter inter-word intervals, since a short inter-word interval would be terminated by the first keypress of an upcoming word before the probe would be played. Action-locked RPs become larger and begin earlier as inter-action intervals increase (Verleger et al. 2016). Our probes might therefore be more likely to interrupt larger RPs than smaller ones. Because shorter inter-word intervals also imply probes following shortly after the preceding action, we also the considered the possible influences of the previous action, as opposed to the upcoming action. However, supplementary analyses suggested this could not explain the association we observed between RP-like potentials and conscious intention (Fig. S9; *Influences of previous actions on probe-locked effects* in *Supplementary Material*). Another possible explanation stems from the idea that shorter and longer inter-word intervals may reflect two different aspects of fluency generation, which Ovando-Tellez et al. (2022) have labeled *cluster* and *switch* respectively. Participants can either generate items drawn from a cluster of close associates (eg frog, toad, newt… for the animal category) or can switch between two clusters that are less close (eg frog, lion…). Switching is thought to recruit additional executive functions, and require additional processing time, compared to clustering (Mastria et al. 2021; Ovando-Tellez et al. 2022). Switching might therefore be over-represented in our probe-locked averages, since these longer switch-related intervals would be disproportionately likely to be interrupted by our probes. These hypotheses could be explored in replication studies, perhaps selecting verbal fluency categories that particularly favor cluster or switch behaviors.

Our probe-locked correlates of prospective intention had a characteristic frontal, topography (Fig. 3B). After excluding the possibility of confounding by eye movements (for detailed analysis, see *Influences of eye movements on probe-locked effects* in *Supplementary Material*), we suggest this anterior focus of our RP-like potentials may indicate a prefrontal contribution of prospective intention. This suggestion can only be tentative, given the poor spatial resolution of EEG, but could potentially be confirmed in future event-related fMRI designs. Alternatively, the cognitive complexity of typing verbal fluency, over and above simple manual keypress actions, may explain this contrast between the traditional fronto-central RP and our more prefrontal RP-like signal. Interestingly, problem-solving studies also show neural activity at prefrontal sites that peaks 1 s or more before action onset (Bieth et al. 2024). Further, prefrontal cortex involvement in voluntary action generation has been shown in studies with both normal adults and patients (Frith et al. 1991; Hunter et al. 2004; Khalighinejad et al. 2016; Rae et al. 2020).

### Methodological considerations & limitations

Capturing the essence of volition in laboratory experiments is difficult, due to the persistent tension between the need for rigorous control on the one hand, and the focus on endogenous, autonomous action on the other (Haggard 2024). Some experiments on volition achieved strong experimental control by focussing on actions that are arbitrary and meaningless (Libet et al. 1983; Matsuhashi and Hallett 2008), while others have endeavored to include reasons, goals and values within the experimental paradigm (Maoz et al. 2019; Seghezzi et al. 2025). To our knowledge, this is the first time that action fluency tasks have been explicitly used to study processes of volition. Our typing-based verbal fluency task emphasizes the internal-generation and spontaneity required by recent definitions of volition (Haggard 2019). Here, the (near) infinity of semantic space is considered as a range of possible actions. The category given to the participant means that only some words/actions are appropriate, and thus makes foraging for suitable words/actions a meaningful and goal-directed cognitive process. Finally, a genuine and readily-reportable subjective experience accompanies the process of determining what action one will make.

Nevertheless, our experimental paradigm may seem new or even strange at first sight, since the voluntary actions occur in the context of a language task. Typing verbal fluency clearly has two aspects: a cognitive process of searching stored lexical items, and a motor process for initiating and executing the action of typing the word. Everyday communication requires that cognitive and motor processes come together, both in free speech production, and in our typing verbal fluency task. Our experimental paradigm draws on suggestions by Lashley (1951) that both cognitive and motor processes are essential to language production. According to Lashley, even “inner speech” involves a covert action generation process that does not reach the threshold in the excitability for overt movements.

Tip of the tongue phenomenon (ToT) provides another interesting example of cognitive and motor processes working closely together in language production. ToT is accompanied by a characteristic experience of conscious effort: one feels that the word one is searching for is “almost there.” Two main theories attempt to explain the mechanism behind ToT: insufficient activation, where target forms and spreading among similar word forms are insufficiently activated, and blocked retrieval, where competitors with phonologically similar words are suppressed (Meyer and Bock 1992; Stasenko and Gollan 2019). The neural correlates of ToT include pupil dilation (Ryals et al. 2021) and suppressed sensorimotor EEG oscillations (Shen et al. 2022)—both of which are also reliable neural correlates of volitional action initiation (Richer and Beatty 1985; Pfurtscheller 1992). For our purposes, a key feature of ToT is the momentary transition between a very definite, and often frustrating initial experience of **not** accessing the motor programme for the target word, and the common experience of finding the word *later*.

Schurger et al. (2012) have recently drawn attention to the effects of biased sampling in investigations of precursor events for volitional action (Schurger et al. 2012; Schurger et al. 2021). They have shown how the practice of action-locked EEG averaging can arbitrarily give the impression of a specific precursor process, such as the RP, as a reliable cause volitional action. Importantly, action-locked averaging cannot determine whether readiness potentials are exclusively associated with impending voluntary action, or whether they in fact happen also at other times. They suggested that the RP might merely be an artifact of averaging a purely stochastic process of accumulating neural noise that serves as the boundary-crossing trigger for voluntary actions in the unnatural experimental conditions where people must freely choose the time of action. Our key inferences in the current study are based on probe-locked analysis, and may therefore avoid some of the specific biases associated with action-locked measures. Moreover, the timing of our probes—though not completely random—should be relatively unbiased with respect to the action. Importantly, our study was not designed to address question about the causal status of the RP. Rather our study aimed to identify whether specific neural precursors were reliably associated with a subjective experience of being about to make a specific action.

We acknowledge that our study has several limitations. First, our mental chronometry approach uses estimates of when a word will be retrieved and an action made, in order to probe intentions. However, our estimates of W^*^ time must be treated with caution for several reasons. (i) A single numerical estimate is typically less informative about psychological processes than a difference between estimates made in contrasting conditions of an experimental design (Haggard and Eimer 1999). (ii) Our W^*^ estimates of awareness latency are calculated relative to a simple *estimate* of likely onset time of actions that were in fact *interrupted by the probe*. The estimate, therefore, cannot be directly related to any objective action event or ground truth. (iii) Further, our use of IWIs for online probe timing means that the estimate of likely action onset is not independent of the preceding action. These factors inevitably limit the precision of our W^*^ estimates, and make it difficult to compare them directly to chronometric estimates using other methods (Libet et al. 1983). Future studies might potentially compare W^*^ time estimates between two conditions of interest, tested in the same participants, since any bias introduced by the estimation process might be common to both conditions.

Second, the number of probes per participant (*M* = 89.04, *SD* = 13.39 in Expt 1, and *M* = 91.04, *SD* = 16.49 in Expt 2) is relatively low, limiting our statistical power to detect a difference between being aware vs. unaware at the time of the probe. Although the number of categories tested far exceeds most verbal fluency tests, and the number of words was generated relatively high, only a small number of inter-word intervals were probed. We reasoned that excessively frequent probing would risk disrupting the cognitive processes of word generation. In particular, the low number of probes limits the possibility of exploratory analysis of possible subsets within our probe-locked EEG data (see *Time course factors* in *Supplementary Material*).

Third, typing verbal fluency involves a combination of a cognitive process of word generation and a more motoric process of manual action preparation. Distinguishing cognitive processes of lexical retrieval from more motoric processes of action preparation is difficult. We have assumed a simple serial model involving search, retrieval, preparation and then action. However, we cannot know which process is active at the time of the probe. Importantly, the word generation process is not stationary. Fluency decays over time, or can show clustering (Mastria et al. 2021; Ovando-Tellez et al. 2022). Our probing algorithm attempted to adjust for the former (though not the latter), but the adjustment may be imperfect. This opens the possibility that awareness of conscious intention may *not only* relate to the stage of preparation for an impending action (Matsuhashi and Hallett 2008), but may *also* be confounded by differences in the cognitive processes of action generation. For example, we found that probes early in the fluency trial were more likely to elicit reports of intention than probes later in the trial (Fig. S8A and B). Could our estimate of neural correlates of conscious intention be confounded by neural correlates of early stage fluency? Our dataset may not have enough probe epochs to assess possible confounds by subdividing and comparing subsets of the data. Further, the precise form of the potential confound is not known, so such analyses could only be exploratory. However, our analyses of EEG spectra and epochs locked to actions, rather than locked to probes, suggests this is not the case (see *Time course factors* in *Supplementary Material*). Further research with larger datasets, and more sophisticated, adaptive probe delivery could provide a stronger test for such confounds.

## Conclusion

To conclude, we have developed a novel paradigm for investigating the neural precursors and subjective correlates of volitional action generation, by combining a typing verbal fluency task with probe-based sampling of conscious experience. Within this paradigm, the experience of becoming aware of a word that one will type next provides a novel, and intuitive operationalization of the experience of conscious intention to initiate volitional action. Importantly, our experimental paradigm gives participants clear reasons for action, and a readily-identifiable, easily-reportable experience of conscious intention, that also resembles the common everyday experience of the right word coming to mind. We suggest that our paradigm overcomes some of the methodological difficulties that have affected previous research on conscious intentions. Using this paradigm, we could reliably identify a transition from no conscious intention to act, to a reportable experience of conscious intention linked to typing a specific word. These reports of conscious intention were necessarily prospective rather than retrospective, because they were evoked by pseudorandom probing during the interval preceding an action, rather than being retrospective reports made subsequent to an action. Our task design then allowed us to investigate EEG correlates of such prospective intentions. We showed that a negative-going potential, reminiscent of the classic RP but with a more anterior bilateral prefrontal topography, was more strongly associated with the presence of prospective intention than its absence. Analysis of LRP tentatively suggested that prospective intention was preceded by a shift of EEG activity towards the cortex contralateral to the hand that would make the action. These results are difficult to reconcile with the view that experiences of conscious intention are merely a retrospective confabulation (Dennett 1993; Wegner 2004). Instead, we suggest that prospective experiences of conscious intention involve, at least in part, a non-inferential readout of the neural precursors of action generation processes.

## Supplementary Material

SupplementaryMaterial_CerCor_Huang_R1_final_bhag108

## Data Availability

Data and codes are publicly available on the Open Science Framework: Huang, Z. (2026, June 26). Action fluency and conscious intention: being aware of what we are about to do. 10.17605/OSF.IO/TMFD4.
