# Supplementary material for: Action fluency and conscious intention: being aware of what we are about to do
Source: Cereb Cortex. 2026 Aug 10;36(7):bhag108. doi: 10.1093/cercor/bhag108 (PMC13456334; doi:10.1093/cercor/bhag108)
Supplement: Supplementary file 1 [file supplementarymaterial_cercor_huang_r1_final_bhag108.docx]

**Supplementary material for**

Action fluency and conscious intention: being aware of what we are about to do

Running title: Action fluency and conscious intention

Zheng Huang 1

Silvia Seghezzi 1, 2

Patrick Haggard 1*

1 Institute of Cognitive Neuroscience, University College London, London, UK

2 Current address: School of Psychological Sciences, Birkbeck, University of London, London, UK

*Corresponding author: Patrick Haggard

Email: p.haggard@ucl.ac.uk

Address: Institute of Cognitive Neuroscience, 17 Queen Square, London, WC1N 3AZ, UK

**Supporting analysis**

***Offline estimation of time of conscious intention*** ***using probe data***

Chronometric measures of conscious intention have been influential, despite the difficulty of interpreting a single numerical estimate (Libet et al., 1983). However, the chronometric measures provided by our probing method could potentially contain an estimation bias, since they are related to an estimate of likely action onset, rather than actual action onset. To assess how well the IWI model captured the data, we report marginal and conditional R-squared for fixed and random effects respectively (see Table S1). In Expt 1, when using participant as the random factor, the mixed model accounted for 22.2% of the variance to fixed effects (marginal R-squared) and 28.0% when random effects were included (conditional R-squared). When using category as the random factor, the model accounted for 23.6% and 30.0% of the variance (marginal and conditional R-squared, respectively). In Expt 2, model using participant as random factor explained 18.0% and 24.0% of variance (marginal and conditional R-squared, respectively), and model using category as random factor explained 17.6% and 25.5% of variance (marginal and conditional R-squared, respectively). Across all four models, the R-squared values indicate that the IWI models accounted for a substantial proportion of variance, suggesting good overall model fit.

Our IWI models include an (exponential) increase in IWI over the course of the 60 s trial. This is only an approximation of what is presumably a more complex non-linear process. It should be noted that these models characterise the overall trajectory of behaviour across the trial, rather than capturing local variations in action patterns, such as clustering behaviour. To quantify the bias in our estimates of IWI for the upcoming action predicted from these models, we selected those actions that were not in fact probed, and then simulated when our algorithm would have *estimated* those actions to occur, treating them as if they had been interrupted by a probe. We could then compare the estimated and *actual* time of these selected actions to estimate the bias of our algorithm. The normal actions used for this simulation were those actions immediately preceding each probe. By performing leave-one-out-cross-validation (LOOCV) for these ‘normal’ actions, we found that our estimations of likely action onset when interrupted by probes were indeed biased: our algorithm estimated that these actions would occur earlier than they actually did occur by 858 ms (95% CI: [705, 1010]) and 660 ms (95% CI: [555, 765]) for Experiments 1 and 2 respectively. This discrepancy between estimated and actual onset time could reflect the very simple model we used to estimate the likely time of the action onset interrupted by probes. However, including further predictors in the estimation model, such as word frequency, and successive semantic distance did not improve model fits.

If we adjust our measures of conscious intention timing by this estimate of bias, we obtain average values of -1881 ms and -1791 ms, respectively for the W* estimate of the time conscious intention in Expts 1 and 2, relative to action onset. The error of these estimates cannot be calculated, due to the unknown nature of variances in the estimation of the next action onset and W* estimation. However, the mean values are comparable to Matsuhashi and Hallett’s previous estimate made using completely random probes (Matsuhashi & Hallett, 2008). Our procedure for adjusting for estimation bias should be treated with caution, because it assumes probed and unprobed intervals are similar, which may be unwarranted. Finally, we note that our probe-locked analyses of neural correlates of prospective intention are independent in principle of biases in action estimation and probe delivery.

***Time course factors***

Verbal fluency tends to decrease over the time course of a single trial. In our paradigm, this might induce an unbalanced sampling, with probes early in a trial being more likely to be associated with reports of conscious intention, and later probes less likely. This trend is shown in sample participants and across all participants in Expts 1 and 2 (Fig. S8*A-B*). Difference in RP-like potentials prior to aware vs unaware probes might therefore be confounded by any differences in EEG between earlier vs later sections of the trial. We therefore compared the global spectral power at FCz in the range 0-30 Hz, and in the time window [-2000 -1000] ms prior to normal action onset (a period when no other events occurred, so EEG spectra should be uncontaminated by event-related potentials), between earlier and later 30 s periods within each trial. This comparison provides a blunt but relevant way of identifying whether the links between conscious intention and RP-like potentials could be an artefact of time-in-trial. No difference was found in global spectral power (*t*(25)=.17, *p*=.86, two-tailed) between earlier and later halves of a trial (Fig. S8*C*).

To examine more directly whether the potentials of interest in this study vary with the phase of the verbal fluency task, we compared RP amplitudes in the [-1000 0] ms window prior to normal action onset between early and late halves of the trial. We chose normal actions where the pre-action epoch was uncontaminated by any other events (such as a probe, or a previous manual action). We separately averaged EEG epochs from the two halves of the trial. No difference was found for the RP amplitudes between the two halves (*t*(25)=-.69, *p*=.50, two-tailed, see Fig. S8*D*).

Importantly, our design includes many more ‘normal actions’ than probed actions. Splitting the probe-locked epochs according to the phase of the verbal fluency task would subdivide an already-small dataset, and reduce statistical power. Therefore, such subset analyses are more appropriate for normal action epochs than for probe-locked epochs.

We have shown that neither the background EEG, nor the pre-action EEG analyses, suggest time-related confounding of our contrast between aware and unaware probes.

***Influences of eye movements on probe-locked effects***

As shown in Fig. 3*B*, the frontal topography of awareness prior to probes raises the possibility of oculomotor confounds. We therefore inspected the correlations between FCz signals and EOGs, and observed how removal of ICA components identified as oculomotor would influence these correlations. If artefact removal is successful, then the correlation between EEG an EOG should fall to a level similar to shuffled-epoch data in which EEG and EOG signals are measured from different epochs, and therefore cannot be driven by a common eye-movement event.

We subtracted, rectified, and integrated (with a 5 Hz low-pass filter) the EOG signals. We found that the correlation between FCz EEG and EOGs significantly reduced from a mean correlation of 0.55 before to 0.38 after ICA removal (*t*(25)=10.91, *p*<.001). To provide a null baseline value for the correlation, we shuffled epochs so as to correlate signals from FCz and EOGs from different epochs. This gave correlations of 0.34 (95% CI: [.33 .34]) and 0.30 (95% CI: [.29 .30]), respectively for before vs. after ICA removal. Even after shuffling epochs, there will be events within epochs which will influence both the brain and the eyes. This will lead to a correlation between EEG and EOG signals even if they come from different epochs, resulting in the residual correlation after shuffling. However, the mean correlation between FCz and EOG signals following ICA removal (r=.38) was comparable to that observed in shuffled epoch data (r=.30), suggesting that EOG signals accounted for minimal variance in FCz activity. Thus, we suggest that ICA removal from our EEG signals was largely successful, and the frontal components associated with awareness of intention are unlikely to reflect eye movement confounds.

***Influences of previous actions on probe-locked effects***

Whereas previous studies of volition involve only a single action epoch per trial, our 60 s verbal fluency trials involve multiple words, and therefore multiple successive actions. We observed significant differences in neural activity during the 1s prior to probes, according to whether those probes elicited aware vs unaware reports. We therefore also considered whether these differences could be related to other confounding events such as typing the final letters of the preceding word. The mean interval between the last keypress of the previous word and probe onset was shorter when participants reported intention at the time of probing (454 ms, *SD*=83 ms) than when they did not (523 ms, *SD*=53 ms) (two-tailed paired *t*-test: *t*(24)=5.12, *p*<.001). Note that this is not inconsistent with our main finding that probes closer to **upcoming action** are more likely to elicit aware than unaware response: the present analysis is locked to the **last** keypress of the **preceding** word. Therefore, some persisting EEG activity associated with previous action could potentially contaminate the apparent link between neural precursor activity for the upcoming word, and conscious intention. To examine this possibility, we adapted an analysis developed by Hackley and Valle-Inclán (1998) which aims to attribute a difference between EEG traces in two conditions to one of two possible events that occur with a variable interval between them. On this logic, if our apparent effects of conscious intention of an impending action in fact reflect some feature of the *preceding* action, then the difference between intention and no-intention neural activities should be stronger when the data is viewed time-locked to the preceding action, than when the same data is viewed time-locked to the probe. The data segment most appropriate for this comparison is the data segment between the previous action and the probe. We found this data segment to have a mean duration of 489 ms. We therefore compared the mean RP-like activity at FCz for aware and unaware responses in a 489 ms window prior to the probe, and additionally in a 489 ms window *following* the last preceding action. The effect of intention was numerically greater for probe-locked epochs (2.62 μV, *SD*=4.58 μV) than for epochs locked to the preceding action (1.18 μV, *SD*=3.72 μV), though the difference did not reach statistical significance (two-tailed paired *t*-test: *t*(24)=-1.67, *p*=.11). Analysis of beta-band amplitudes showed that the effect of intention was significantly larger for probe-locked (.064 μV, *SD*=.150 μV) than previous-action-locked data (.006 μV, *SD*=.121 μV) (two-tailed paired *t*-test: *t*(24)=2.45, *p*=.02). We thus found no consistent evidence that the probe-locked differences we observed are in fact a residual consequence of some feature of the previous action (see Fig. S9).

***Localising prospective intention in the brain***

The EEG topography for probe-locked analysis (Fig. 3*B*) suggested that the differences between intention and no intention conditions may be most strongly present at frontal sites. This raises the exploratory question whether more frontal site (e.g., Fz) would be more appropriate as the centre of the effect we have observed. We exploratorily analysed data from electrode Fz to test this hypothesis. Additional epoch boundary rejection was done with Fz added as a channel of interest, and the same analysis pipeline was performed. The result showed a larger RP-like activity (one-tailed paired *t*-test: *t*(25)=3.09, *p*=.002, Cohen’s *d*=.61), as well as a marginally lower mean beta amplitude (one-tailed paired *t*-test: *t*(25)=-1.70, *p*=.051, Cohen’s *d*=-.33) prior to probes when participants reported conscious intention, compared to when they did not. These effect sizes are comparable to the effect sizes at FCz, which had been pre-selected as the main channel of interest, because it is classically associated with the readiness potential (Khalighinejad et al., 2018; Takashima et al., 2020; Travers et al., 2020). We therefore are open to the possibility that the current prospective intention effect has a more prefrontal focus.

**Supporting material**

List of verbal fluency categories used in Experiments 1 and 2.

ARTWORK TYPES

BIRDS

BUILDING MATERIALS

CLOTHING ITEMS

COLOURS

DRINKS

FISHES

FLOWERS

FRUITS

FURNITURE

HERBS & SPICES

ILLNESSES & DISEASES

JOBS

KITCHEN UTENSILS

METALS

MUSICAL INSTRUMENTS

SPORTS

SUBJECTS STUDIED AT SCHOOL

TOOLS

TOYS

TRANSPORTATION MEANS

VEGETABLES

WEAPONS

WEATHER EVENTS

**Figures**

**Fig. S1. Time of intention awareness using participant as a random factor in Experiment 1 (n=23). A. Individual participant fits.** Mixed-model logistic regression fits are shown for each participant (first-level model fits). Time 0 is the estimated onset of the next word. Each datapoint represents a single probe, eliciting a response indicating awareness of intention to type the next word (“Yes”), or absence of intention (“No”). Each curve is from a single participant. The time that each curve crosses the awareness threshold provides an estimate of conscious intention time (W* time). **B. Distribution of individual participant W* estimates.** The histogram shows participants are most likely to have a W* estimates between -2 s and -1 s before estimated onset of the next word.

**Fig. S2. Time of intention awareness using category as a random factor in Experiment 1 (n=24 categories). A. second-level model fit.** Time 0 is the estimated onset of the next word. Each datapoint represents a single probe, eliciting a response indicating awareness of intention to type the next word (Blue), or absence of intention (Orange). The time that the curve crosses the awareness threshold provides an estimate of conscious intention time (W* time). **B. Individual category fits.** Mixed-model logistic regression fits are shown for each category (first-level model fits). **C. Distribution of category W* estimates.** The histogram shows that categories often have a W* estimate between -2 s and -1 s before estimated onset of the next word.

**Fig. S3. Time of intention awareness using participant as a random factor in Experiment 2 (n=28). A. Individual participant fits.** Mixed-model logistic regression fits are shown for each participant (first-level model fits). Time 0 is the estimated onset of the next word. Each datapoint represents a single probe, eliciting a response indicating awareness of intention to type the next word (“Yes”), or absence of intention (“No”). Each curve is from a single participant. The time that each curve crosses the awareness threshold provides an estimate of conscious intention time (W* time). *Two participants whose curves which did not cross the threshold were excluded in the calculation of W* estimates, but their curves are shown here.* **B. Distribution of individual participant W* estimates.** The histogram shows participants are most likely to have a W* estimates between -2 s and -0.5 s before estimated onset of the next word.

**Fig. S4. Time of intention awareness using category as a random factor in Experiment 2 (n=24 categories). A. second-level model fit.** Time 0 is the estimated onset of the next word. Each datapoint represents a single probe, eliciting a response indicating awareness of intention to type the next word (Blue), or absence of intention (Orange). The time that the curve crosses the awareness threshold provides an estimate of conscious intention time (W* time). **B. Individual category fits.** Mixed-model logistic regression fits are shown for each category (first-level model fits). **C. Distribution of category W* estimates.** The histogram shows participants are most likely to have a W* estimates between -2 s and -1 s before estimated onset of the next word.

**Fig. S5. Neural activity before normal (unprobed) action onset (n=26). A. Topographic plot of average EEG amplitude.** The plot shows average EEG amplitude interpolated across the scalp in a time window of -1000 ms to 0 ms relative to normal action onset where no probe interrupted the action. **B. Grand-average EEG beta-band amplitudes at electrode FCz.** The plot shows EEG amplitude in a time window of -800 ms to 0 ms relative to normal action onset where no probe interrupted the action.

**Fig. S6. Predicted effects of realigning lateralized-readiness-potential-like (LRP-like) activity.** Desynchronization or resynchronization can change the *sign* of the LRP-like potential calculated by double subtraction. The left-hand column shows illustrative traces (not data) action-locked at sites contralateral and ipsilateral to movement. In the second column, these data are realigned to realignment points which hypothetically occur at different times prior to action (comparison across rows). Each channel is baseline-corrected at the time of the realignment point (column 3), and the contralateral-ipsilateral difference is calculated. Depending on the time of the realignment point relative to the hypothesized action, the predicted LRP-like potential may be either positive or negative (see arrows in column 4). If the realignment point is close to action onset, the mean LRP-like activity would be mostly positive (white arrow). As the realignment point moves earlier relative to action onset, the mean LRP-like activity will tend to decrease and become negative (black & red arrows). If the realignment point happens before LRP onset (lower row), no LRP-like activity would be seen prior to the realignment point (blue arrow). Note that all graphs use the ‘negative up’ convention for plotting EEG.

**Fig. S7. Distributions of key timing variables. A. Distribution of inter-word intervals preceding normal action in Expts 1 (left) and 2 (right).** Distribution of inter-word intervals (grey bars) with kernel fits (black lines) are shown. **B. Distribution of abnormal probe responses in Expts 1 (left) and 2 (right).** Participants were instructed to respond to the probe and accompanying question about awareness by typing “Yes” or “No”. Occasionally, participants responded by pressing other keyboard keys: here we consider how to classify these responses. In Expt 1, probe responses that were not classifiable as “Yes” and “No” were plotted with kernel fit. In Expt 2, we assessed whether what participants typed could be considered a ‘finger error’ – i.e., the participant attempted to type “Yes” or “No”, but pressed the wrong keyboard keys. Probe responses that contained finger errors are shown in purple. The remaining probe responses were judged unclassifiable (green). Distribution of abnormal probe responses were plotted with kernel fits. Note many unclassified responses occurring shortly after probes. These are likely to be words generated in the verbal fluency task which had passed a point of no return by the time the probe occurred – participants’ typing actions would then correspond to the verbal fluency task, rather than typing responses to the post-probe questions.

**Fig. S8. Time course of verbal fluency related factors. A. Distribution of probes in sample participants.** Frequency plots show the time of occurrence within the 60s trial of probes associated with aware vs. unaware responses in illustrative participants from Expts 1 (left) and 2 (right). Raster-type diagrams below show the time of probes on each trial. **B. Distribution of probes across participants.** Frequency plots and raster-type diagrams as in panel A are shown. The plots are drawn from pooled participant data for Expts 1 (left) and 2 (right). **C. Power spectral density of background EEG.** Power spectral density was calculated for background EEG in early (yellow) and late (purple) phase of a trial. **D. EEG amplitudes around time of normal action.** Only action epochs uncontaminated by other events (probes, other actions) were included. EEG amplitudes (±*SEM*) locked to action in early (yellow) and late (purple) phase of a trial are shown. Note similar action-related EEG activity at early and late phases of the verbal fluency trial.

**Fig. S9. EEG signals at electrode FCz locked to *previous* action (n=26). A. EEG amplitude (±*SEM*) around the time of the previous action.** Mean EEG amplitudes from -500 ms to 500 ms relative to previous action onset are shown. The difference between aware and unaware trials is numerically smaller in previous-action-locked than in probe-locked analyses, but not significantly so. **B. Grand-average EEG beta-band amplitude locked to the *previous* action before probe in aware and unaware conditions (left) and their difference (right).** The grand average interval between the previous action onset and the subsequent probe is 489 ms. During this interval, EEG signals are separated according to the awareness judgement made to subsequent probe. The difference between aware and unaware trials is significantly smaller in previous-action-locked than in probe-locked analyses. Note that there is a clear edge effect due to the frequency transformation at the end of the epoch, but this is comparable across conditions, and does not appear in the difference plot.

**Fig. S10. Amplitude of an LRP-like potential related to prospective intention in participants using average reference (n=20).** Probe-locked LRP-like amplitude (±*SEM*) was calculated for the average reference subset, using the same method as for the full dataset (Eimer, 1998), at electrodes C3 and C4, from -1000 ms to 0 ms relative to probe onset. † indicates trend-level significance level of .05<*p*<.10, one-tailed.

**Fig. S11. EEG beta-band amplitudes (13-30 Hz) at electrodes C3 (top) and C4 (bottom) before probe onset (n=26).** The plots show mean beta-band EEG amplitude when the first letter of the upcoming word would be typed by left or right hand, averaged across a time window from -800 ms to 0 ms relative to probe onset where conscious intention report was obtained.

**References for Supporting Information**

Hackley, S. A., & Valle-Inclán, F. (1998). Automatic alerting does not speed late motoric processes in a reaction-time task. *Nature*, *391*(6669), 786-788. <https://doi.org/10.1038/35849>

Khalighinejad, N., Schurger, A., Desantis, A., Zmigrod, L., & Haggard, P. (2018). Precursor processes of human self-initiated action. *NeuroImage*, *165*, 35-47. <https://doi.org/https://doi.org/10.1016/j.neuroimage.2017.09.057>

Libet, B., Gleason, C. A., Wright, E. W., & Pearl, D. K. (1983). Time of conscious intention to act in relation to onset of cerebral activity (readiness-potential). The unconscious initiation of a freely voluntary act. *Brain*, *106*(3), 623. <https://doi.org/10.1093/brain/106.3.623>

Matsuhashi, M., & Hallett, M. (2008). The timing of the conscious intention to move. *European Journal of Neuroscience*, *28*(11), 2344-2351. <https://doi.org/10.1111/j.1460-9568.2008.06525.x>

Takashima, S., Ogawa, C. Y., Najman, F. A., & Ramos, R. T. (2020). The volition, the mode of movement selection and the readiness potential. *Experimental Brain Research*, *238*(10), 2113-2123. <https://doi.org/10.1007/s00221-020-05878-9>

Travers, E., Khalighinejad, N., Schurger, A., & Haggard, P. (2020). Do readiness potentials happen all the time? *NeuroImage*, *206*, 116286. <https://doi.org/https://doi.org/10.1016/j.neuroimage.2019.116286>

**Table S1.** *Model fitting diagnostics for IWI mixed-effects model*

|  | **Random factor** | **Marginal R-squared (%)** | **Conditional R-squared (%)** |
| --- | --- | --- | --- |
| **Expt 1** | Participant | 22.2 | 28.0 |
|  | Category | 23.6 | 30.0 |
| **Expt 2** | Participant | 18.0 | 24.0 |
|  | Category | 17.6 | 25.5 |
